# The Impact of Diet Quality on COVID-19 Severity and Outcomes—A Scoping Review

**DOI:** 10.1007/s13668-025-00618-3

**Published:** 2025-02-01

**Authors:** Athina Tassakos, Alanna Kloppman, Jimmy Chun Yu Louie

**Affiliations:** Discipline of Dietetics, Department of Allied Health, School of Health Sciences, Swinburne University of Technology, SPW Building, 1 John St, Hawthorn, VIC Australia

**Keywords:** COVID-19, Dietary pattern, Symptom severity, Immune system

## Abstract

**Purpose of Review:**

The COVID-19 pandemic, caused by SARS-CoV-2, has highlighted the potential role of nutrition in modifying disease susceptibility and severity. This review aims to systematically evaluate the current evidence on associations between dietary patterns, assessed using diet quality scores (DQS), and COVID-19 severity and outcomes.

**Recent Findings:**

A comprehensive literature search identified 15 studies across diverse populations. Prospective cohort studies generally found higher diet quality associated with lower COVID-19 infection rates. Case–control studies consistently showed reduced odds of COVID-19 infection and severe illness with adherence to anti-inflammatory dietary patterns, particularly the Mediterranean diet. Cross-sectional data revealed associations between higher DQS and reduced COVID-19 symptom burden and improved prognostic biomarkers. An ecological study demonstrated inverse relationships between national-level diet quality and COVID-19 caseloads. Mediterranean, Dietary Approaches to Stop Hypertension (DASH), and plant-based diet scores were notably predictive of favourable outcomes, even after adjusting for confounders. Conversely, consumption of processed foods high in saturated fats, sugars, and additives was linked to increased COVID-19 complications. Despite these findings, research gaps remain, including the impacts of specific dietary components, effect modifiers across populations, and establishing causality through interventional trials.

**Summary:**

This review highlights the observational evidence supporting the potential integration of optimal nutrition into pandemic preparedness strategies. Further research is needed to strengthen these findings and inform evidence-based dietary recommendations for COVID-19 prevention and management.

## Introduction

The emergence of severe acute respiratory syndrome coronavirus 2 (SARS-CoV-2) in late 2019 precipitated the global COVID-19 pandemic, straining healthcare systems worldwide [1]. As of June 2023, over 700 million infections and 7 million deaths have occurred globally [2]. SARS-CoV-2 is primarily transmitted through respiratory droplets, with the viral spike protein binding to angiotensin-converting enzyme 2 (ACE2) receptors throughout the body [3]. This triggers an excessive immune response and cytokine storm, causing systemic inflammation and tissue damage [3]. While many cases are mild, some progress to severe complications requiring hospitalization [4], with elevated risks among older people and those with underlying conditions [5].

Emerging research indicates diet quality may influence COVID-19 vulnerability through modulating inflammatory pathways and immunity [5, 6]. Diets rich in bioactive compounds like polyphenols and antioxidants, such as the Mediterranean diet with abundant fruits, vegetables, whole grains, legumes, nuts, and anti-inflammatory unsaturated fats, exert systemic anti-inflammatory effects capable of mitigating cytokine-mediated injury [7, 8]. Conversely, dietary patterns characterized by excessive processed foods, refined carbohydrates, and pro-inflammatory lipids may amplify dysfunctional immune activation and metabolic disturbances, potentially exacerbating COVID-19 severity [9].

While preliminary studies suggest healthier diets may reduce COVID-19 severity, considerable gaps remain regarding long-term impacts, roles of specific foods or nutrients, consistency across populations, and mechanisms interfacing with SARS-CoV-2 pathogenesis. This scoping review aims to systematically map existing evidence evaluating associations between dietary patterns, diet quality, and COVID-19 symptom severity/outcomes to identify research priorities, inform paradigms for future investigations into nutrition’s role in modulating COVID-19, and guide public health policies/interventions empowering dietary choices to bolster immunocompetence and optimize healthcare resources.

## Methods

### Study Design and Search Strategy

This scoping review follows the methodology outlined in the Preferred Reporting Items for Systematic Reviews and Meta-Analyses Extension for Scoping Reviews (PRISMA-ScR) guidelines [10]. As outlined in the Joanna Briggs Institute Manual for Scoping Reviews, the scoping review design was chosen to systematically search, evaluate and synthesize current research on associations between dietary patterns, assessed using diet quality scores and COVID-19 symptom severity outcomes [11].

A systematic search was conducted in June 2023 using MEDLINE, PubMed, Web of Science, Scopus and CINAHL databases. The search combined relevant terms related to diet quality scores AND COVID-19 symptom severity using Boolean operators and parentheses. The keywords used in the search strategy were as follows: (COVID-19 OR COVID OR coronavirus AND diet pattern OR diet intake OR diet score OR diet index OR diet quality score OR healthy eating index OR healthy eating index for Australian adults OR Canadian healthy eating index OR the US healthy eating index OR healthy food index OR diet quality index OR Aussie-diet quality index OR Danish diet quality index OR Chinese diet quality index OR diet quality index Swedish nutrition recommendations OR healthy diet indicator OR Mediterranean diet score OR Nordic food index OR dietary approaches to stop hypertension OR UK nutrient profile score OR German food pyramid index OR Baltic sea diet score OR total diet score OR dietary guideline index AND reduced symptoms OR symptoms OR severity of symptoms). Additionally, a hand search of the reference lists of relevant studies was performed to identify further eligible studies that may have been missed in the initial database search. An additional search specifically targeting the Global Diet Quality Score (GDQS) [12] was conducted in Sep 2024; however, this search returned no relevant results.

### Study Inclusion Criteria

The included studies met the following criteria: (1) published in English between March 2020 and May 2023; (2) evaluated associations between diet quality and COVID-19 symptom severity outcomes; (3) utilized a pre-defined DQS to assess diet quality. Reviews, case studies, protocols, and unpublished data were excluded.

### Screening Process

Articles retrieved through the systematic search were imported into EndNote, and duplicates were removed. Two researchers (AK and AT) then independently evaluated titles, abstracts and full texts stepwise against the pre-defined inclusion/exclusion criteria. Conflicts were resolved through discussion at each stage. The study selection process was documented using a PRISMA flowchart [11].

### Data Extraction

A customized data extraction form was developed and iteratively refined to obtain key data from included studies. Extracted data encompassed author names and publication year, study location, timeline, design and sample population; diet assessment method and DQS utilized; COVID-19 symptom severity measures; key findings related to diet quality and symptom outcomes, including effect sizes and statistical significance where applicable.

### Use of Large Language Models

ChatGPT 3.5 was used to improve the manuscript text drafted by the authors, using the prompt “improve the text below for clarity, coherence, flow and grammar for an academic audience”. The senior author (JCYL) has reviewed the output for accuracy and takes full responsibility for the content presented.

## Results

### Study Characteristics

**Fig. 1:**
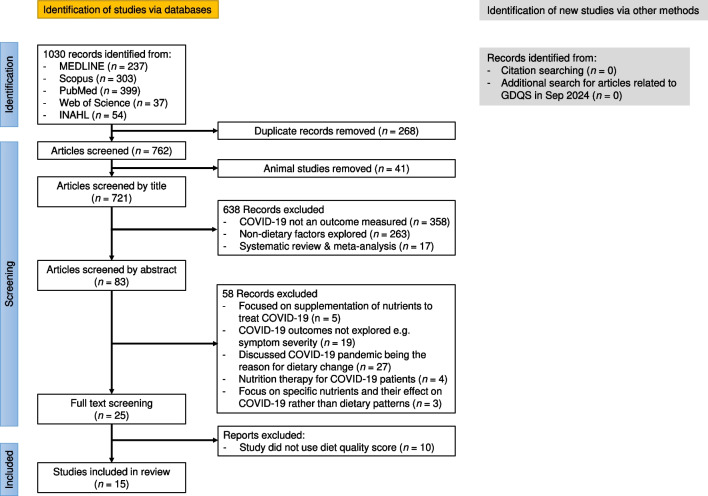
Preferred Reporting Items for Systematic Reviews and Meta-Analyses (PRISMA) flow diagram summarizing the process of screening and identification of the literature in the scoping review. GDQS, Global Diet Quality Score

The systematic literature search identified 1,030 records, of which 15 studies met all eligibility criteria and were included in this scoping review (Fig. 1). Table 1 summarizes 15 studies examining the relationship between dietary quality and COVID-19 outcomes. There were five prospective cohort studies, four case–control studies, five cross-sectional studies, and one ecological study. Sample sizes ranged considerably from 100 to 592,571 participants. Most studies had a predominance of female participants and included adults over 18 years old, with six studies specifically focusing on older adults (mean age > 50 years). Geographically, the studies were conducted in various regions across Asia, Europe, and North America.

**Table 1:** Summary of the included studies

Study	Population	Diet quality measures and assessment	Key findings	**Limitations**
*Prospective cohort studies*				
Deschasaux-Tanguy et al. [15]	*n* = 7,766 adults 70.3% women Mean age: 60.3 y France	AHEI-2010, sPNNS-GS2 3 × dietary records (2 week days + 1 weekend day), collected at least twice	No link between overall diet quality and likelihood of COVID-19 infection, but nutrients such as vitamin B_9_, C, K, fibre associated with decreased odds. Higher dairy/calcium linked to increased odds	Cohort study design unable to infer causation; self-report bias; antibody test limitations, missing data like race/supplements, differences between groups
Merino et al. [6]	*n* = 592,571 adults 68.2% women Age 18 y + (52.8% 55 y +) UK & US	hPDI 27-item semi-quantitative FFQ	Higher hPDI was associated with 18% and 41% lower risk of COVID-19 and severe COVID-19 respectively. Those of high socioeconomic deprivation with low hPDI had a 47% increased risk of COVID-19, compared with those of low socioeconomic deprivation with high hPDI	Cohort study design unable to infer causation; recall bias; self-report bias; brief FFQ reducing precision
Perez-Araluce et al. [46]	*n* = 9,677 adults 62.3% women Mean age: 52.6 y Spain	MDS 136-item semi-quantitative FFQ	Every 2-point higher MDS was linked to 26% lower COVID-19 likelihood in non-healthcare workers only	Cohort study design unable to infer causation; recall bias; self-report bias; residual confounding
Perez-Araluce et al. [47]	*n* = 9,485 adults 62.3% women Mean age: 52.9 y Spain	MDS 136-item semi-quantitative FFQ	Higher Mediterranean diet linked to lower likelihood of COVID-19 in non-healthcare professionals only. Yogurt and whole dairy reduced risk. Higher olive oil increased COVID-19 likelihood in the overall study population (which includes healthcare workers). No significant between MDS and COVID-19 symptoms or severity was observed	Cohort study design unable to infer causation; recall bias; self-report bias; attrition bias from participants dropping out over time
Wang et al. [16]	*n* = 1,981 female nurses Mean age: 65.9 y US	AHEI-2010 152-item semi-quantitative FFQ	Healthier diet alone was not significantly associated with lower post-COVID-19 conditions; however, individuals who had higher number of healthy lifestyle factors (which includes healthy diet) had lower risk of post-COVID-19 conditions	Cohort study design unable to infer causation; recall bias; self-report bias; findings limited to middle-aged white female nurses; COVID-19 strain differences
Yue et al. [14]	*n* = 42,935 adults 90% women Age 55–99 y US	AHEI-2010, AMED, EDIH, EDIP 150 + items Semi-quantitative FFQs	Healthier dietary patterns identified by all 4 dietary quality measures were associated with lower SARS-CoV-2 infection likelihood. Individuals with healthier diets were less likely to contract severe COVID-19 infection or require hospitalization, but these associations became non-significant after accounting for BMI/comorbidities	Cohort study design unable to infer causation; recall bias; self-report bias; selection bias from only including those who completed most recent survey; lack of information regarding fatal cases
*Case–control studies*				
El Khoury, Julien [17]	*n* = 399 adults 69.9% women Age 21 y + (10.8% > 50 y) Lebanon	MedDiet score by Panagiotakos et al*.* [13] Mediterranean diet questionnaire and 16-item semi-quantitative FFQ	Higher Mediterranean diet adherence was associated with lower odds of COVID-19 infection; but not burden of COVID-19	Case–control design unable to infer causation; recall bias; self-report bias; small sample; crude dietary assessment
Firoozi et al. [48]	*n* = 455 adults 54.7% women Mean age ~ 40 y Iran	E-DII 116-item semi-quantitative FFQ	Higher dietary inflammatory potential (higher E-DII score) was associated with a near tripled COVID-19 risk	Case–control design unable to infer causation; recall bias; self-report bias; relatively small case group of 133
Khorasanchi et al. [49]	*n* = 240 adults 44.2% women Mean age: 58.9 y Iran	DASH 68-item semi-quantitative FFQ	Higher DASH adherence inversely associated with depression, anxiety and stress in recovered COVID-19 patients. Higher vegetables, fruit, nut, legume, whole grain tied to lower depression, anxiety or stress	Case–control design unable to infer causation; recall bias; self-report bias; small sample
Kim et al. [19]	*n* = 2,884 healthcare workers 27.5% women Mean age: 48 y USA, UK, Spain, Italy, France and Germany	Plant-based diets; Plant-based diets or pescatarian diets; Low carbohydrate, high protein diets Self-reported type of diet for the past year	Plant-based diets associated with 72% lower odds of moderate-to-severe COVID-19 *vs*. non-plant based; Including pescatarian diet onto Plant-based diets attenuated the association by 13%points. Low carbohydrate, high protein diets were not associated with moderate-to-severe COVID-19. No link to infection risk/symptom duration	Case–control design unable to infer causation; self-report bias; diet definition variability; selective sampling
*Cross-sectional studies*				
Bakırhan et al. [22]	*n* = 100 adults 51% women Mean age: 32.3 y Turkey	MEDAS, DASH, HEI-2015 24-h food recall (HEI-2015); MEDAS questionnaire; intake frequencies of DASH foods from face-to-face interviews	Poor diet quality associated with increased obesity markers in COVID-19 group. Higher diet scores predicted lower CRP levels. Higher fruit intake was associated with lower Troponin-I	Cross-sectional design unable to infer causation; recall bias; very small sample
Merino et al. [6]	*n* = 3,947 adults 55.7% women Mean age: 44.4 y Vietnam	HES-5 HES-5 questionnaire on 5 food groups	Higher HES-5 and vegetable, fruit, fish intake were associated with lower likelihood of suspected COVID-19 symptoms. Those with moderate or high HES-5 and physically active had lower likelihood of suspected COVID-19 symptoms	Cross-sectional design unable to infer causation; recall bias; HES-5 without portion size information
Tavakol et al. [21]	*n* = 206 COVID-19 patients 57.8% women Mean age: 40.9 y Iran	Consumption frequencies of fish, poultry, meat, milk and dairy products, fruit, vegetables, tea, confectionaries and sugar, sausages, fast foods, salad dressings, butter or cream with breakfast, and cheese 16-item food intake questionnaire	Lower poultry, fruit, and higher tea intake associated with more severe COVID-19. Overall healthier diets linked to lower severity	Cross-sectional design unable to infer causation; recall bias; small sample; racial/dietary homogeneity; selection bias excluding severe cases; non-validated and crude dietary assessment
Zargarzadeh et al. [18]	*n* = 250 patients recovered from COVID-19 52.4% women Mean age: 44.1 y Iran	MDS 168-item web-based semi-quantitative FFQ	Higher MDS was associated with 77% lower odds of severe COVID-19; and inversely associated with a variety of COVID-19 symptoms. Those with a higher MDS had lower inflammatory biomarkers e.g., C-reactive protein and erythrocyte sedimentation rate	Cross-sectional design unable to infer causation; Recall bias, especially overweight/obese underreporting intake; FFQ covered past year pre-diagnosis; No information on supplement intake
*Ecological study*				
Greene et al. [8]	17 regions in Spain and 23 OECD countries*	MAI Household food consumption for Spain; FAO food balance sheet for OECD countries	Higher Mediterranean diet adherence associated with fewer COVID-19 cases and deaths in Spain and the 23 OECD countries studied	Ecological design does not allow direct examination of the relationship between exposure(s) and outcome(s); and is unable to infer causation

AHEI-2010, Alternative Healthy Eating Index 2010; AMED, Alternative Mediterranean Diet Score; DASH: Dietary Approaches to Stop Hypertension; EDIH, Empirical Dietary Index for Hyperinsulinaemia; E-DII: energy-adjusted dietary inflammatory index; EDIP, Empirical Dietary Inflammatory Pattern; FFQ: Food Frequency Questionnaire; HEI-2015: Healthy Eating Index 2015; HES-5; Healthy Eating Score questionnaire with five items; hPDI: Healthy Plant-Based Diet Index; MAI: Mediterranean Adequacy Index; MDS: Mediterranean Diet Score; MEDAS: Mediterranean Diet Adherence Screener; OECD: Organisation for Economic Co-operation and Development; sPNNS-GS2: simplified Programme National Nutrition Sante-guidelines score 2

^*^The OECD countries included were: Australia, Austria, Canada, Chile, Denmark, Finland, France, Germany, Greece, Hungary, Iceland, Ireland, Israel, Italy, Japan, Norway, Portugal, Spain, Sweden, Switzerland, Turkey, United Kingdom, United States

### Dietary Quality Measures

A variety of dietary quality measures were utilized across the studies, with the most common being the Alternate Healthy Eating Index 2010 (AHEI-2010, *n* = 3 studies), and the Mediterranean Diet Score (MDS, *n* = 3 studies). Other measures (all *n* = 1 study) included the Alternative Mediterranean Diet Score (AMED), Dietary Approaches to Stop Hypertension (DASH), Empirical Dietary Index for Hyperinsulinemia (EDIH), Empirical Dietary Inflammatory Pattern (EDIP), energy-adjusted Dietary Inflammatory Index (E-DII), Healthy Eating Index 2015 (HEI-2015), Healthy Eating Score with five items (HES-5), Healthy Plant-Based Diet Index (hPDI), plant-based or pescatarian diets, low carbohydrate high protein diets, Mediterranean Diet Adherence Screener (MEDAS), Mediterranean Adequacy Index (MAI), Mediterranean Diet Score by Panagiotakos et al. [13] (MedDiet Score), simplified Programme National Nutrition Sante-guidelines score 2 (sPNNS-GS2), and consumption frequencies of key food groups. While heterogeneous, these diet quality assessment tools share similar components, evaluating relative consumption of recommended food groups, micronutrients, and macronutrients and avoiding limiting nutrients or food constituents.

### COVID-19 Outcomes Assessed

The outcomes examined were COVID-19 infection risk (*n* = 9 studies), severe COVID-19 illness (*n* = 7 studies), COVID-19 symptoms (*n* = 4 studies), post-COVID conditions (*n* = 1 study), inflammatory biomarkers (*n* = 2 studies), and COVID-19 cases/deaths at the population level (*n* = 1 ecological study).

### Association between Diet Quality and COVID-19

Most prospective cohort studies found that higher overall diet quality was associated with lower risks of contracting COVID-19 infection. For instance, Merino et al. [6] reported that higher hPDI scores were linked to 18% and 41% lower risks of COVID-19 infection and severe illness, respectively. Yue et al. [14] found that healthier dietary patterns defined by AHEI, AMED, EDIH, and EDIP were tied to lower SARS-CoV-2 infection likelihood. However, some studies, such as Deschasaux-Tanguy et al. [15], found no overall link between diet quality and COVID-19 infection, though certain nutrients like vitamins and fibre were associated with decreased odds. Additionally, Wang et al. [16] reported that while diet alone was not significantly related to post-COVID conditions, individuals with multiple healthy lifestyle factors, including diet, had lower risks.

Case–control studies generally aligned in showing that adherence to healthy dietary patterns like the Mediterranean diet was associated with lower COVID-19 risk and severity. El Khoury, Julien [17] found higher Mediterranean diet adherence was linked to lower odds of COVID-19 infection in Lebanon, while Zargarzadeh et al. [18] reported that higher MDS was tied to 77% lower odds of severe COVID-19 among recovered Iranian patients. Plant-based diets were also highlighted, with Kim et al. [19] finding they were associated with 72% lower odds of moderate-to-severe COVID-19 among healthcare workers across multiple countries.

Cross-sectional studies provided further evidence that higher diet quality was associated with lower likelihood and severity of COVID-19 symptoms and improved inflammatory biomarkers. Nguyen et al. [20] showed that higher HES-5 scores were linked to lower suspected COVID-19 symptom risk in Vietnam. Similarly, Tavakol et al. [21] found that overall healthier diets were associated with lower COVID-19 severity among Iranian patients. Two studies [18, 22] also reported that higher diet quality predicted lower inflammatory markers such as C-reactive protein levels.

At the population level, the ecological study by Greene et al. [8] found that higher adherence to the Mediterranean diet, measured by the MAI, was associated with fewer COVID-19 cases and deaths across 17 regions in Spain and 23 OECD countries.

## Discussion

This scoping review synthesizes the rapidly accumulating evidence linking diet quality to COVID-19 susceptibility, severity, and recovery trajectories. Across 15 studies covering a range of geographical and demographic populations, adherence to high-quality dietary patterns abundant in fruits, vegetables, whole grains, legumes, nuts, seeds, and healthy fats emerged as a prominent predictor of reduced risks for contracting COVID-19 infection and experiencing severe clinical presentations. Dietary patterns epitomized by the Mediterranean, DASH, and plant-based eating styles surfaced as protective, likely attributable to their potent anti-inflammatory, antioxidant, and immunomodulatory properties conferred by nutrient-dense food profiles. Notably, these protective associations extended to general community populations and high-risk frontline groups like healthcare workers routinely exposed to occupational viral threats, underscoring the fundamental role of optimal nutrition in enhancing physiological resilience. In stark contrast, chronic consumption of highly processed, calorie-dense, yet nutrient-depleted modern industrial food products were unequivocally and strongly implicated in precipitating adverse COVID-19 prognostic trajectories marked by escalating severity and complications.

These findings reinforce and extend the well-established mechanistic underpinnings demonstrating the profound influence of nutrition on respiratory health resilience and immunocompetence against viral and other pathogens [23–25]. At the molecular level, diets replete with bioactive compounds can enhance viral resistance by judiciously modulating inflammation pathways [26], optimizing immune cell function spanning both innate and adaptive responses [27], and fortifying epithelial barriers to mitigate initial infection susceptibility [28, 29]. Specific nutrients like polyphenols, vitamins A, C, D, E, and essential minerals such as zinc and selenium have been implicated in regulating cytokine cascades [30–32], supporting lymphocyte proliferation and activity [33, 34], and bolstering antioxidant defenses [31] – all critical components of an integrated anti-viral response [34]. Synergistic interactions among these dietary components may confer additive or even multiplicative advantages beyond the isolated effects of single nutrients.

An intriguing intersection between diet quality, nutritional status, and anthropometric indices further emerged, underscoring the intricate interplay among these factors in modulating COVID-19 vulnerability. The well-documented heightened COVID-19 risks conferred by obesity appear partly attributable to the insidious effects of underlying metabolic dysfunction manifesting as chronic low-grade inflammation and cytokine derangements – disturbances that may render obese individuals particularly susceptible to viral pathogens capable of precipitating cytokine storm syndromes [35–37]. Among COVID-19 patients requiring hospitalization, elevated BMI was consistently associated with more protracted clinical courses, exacerbated complications, and escalated mortality [38, 39]. However, a particularly interesting and important observation was that individuals with higher adiposity classifications appeared to benefit most profoundly from improvements in dietary quality, suggesting that optimal nutrition could mitigate severity irrespective of baseline weight status.

Recent systematic and scoping reviews have highlighted the importance of diet quality for overall health and nutritional status during the COVID-19 pandemic [40, 41]. Although these reviews examine associations between dietary patterns and general health, there remains limited understanding of how diet specifically impacts COVID-19 severity or outcomes.

The literature underscores the role of nutrient-dense, minimally processed diets in promoting health [42]. However, there is insufficient evidence directly linking dietary components or patterns to the severity of COVID-19 or its clinical outcomes. This gap presents a critical area for further investigation. Understanding the potential connections between diet and COVID-19 outcomes could guide the development of targeted nutritional interventions and public health strategies relevant to pandemic response. Moreover, the interactions among various dietary components warrant closer examination in the context of COVID-19. Nutrient profiling and pathway analyses could help identify protective or risk-modifying effects of specific nutrients or dietary patterns on the severity of the disease.

Additionally, further research is needed to explore the role of potential effect modifiers and differential susceptibilities across population subgroups. While some studies have focused on diet quality during the pandemic in particular regions [43] and age groups [41], more work is required to assess how dietary factors might influence COVID-19 outcomes across diverse demographics. Age, ethnicity, socioeconomic status, pre-existing conditions, and baseline nutritional status are all factors that could play a role. Understanding these variables could help shape dietary guidance for high-risk populations and address the disparities in food access and diet quality that have worsened during the pandemic.

Finally, the long-term effects of diet on COVID-19, particularly in the context of emerging SARS-CoV-2 variants with different mutagenic profiles and transmissibility, remain unclear. Research into how sustained dietary adherence or nutritional interventions implemented at various stages of COVID-19 might affect outcomes is needed. Clarifying the timing and duration of dietary modifications necessary to achieve prophylactic or therapeutic benefits is another area that requires exploration. Addressing these questions through well-designed prospective studies will strengthen the evidence base and support the use of diet as a potential tool in enhancing resilience against emerging viral threats.

Strengths of our scoping review include adherence to established PRISMA-ScR guidelines [10] for standardized conduct and reporting of scoping reviews; incorporation of varied diet quality measures that expanded the comprehensiveness of capturing relevant dietary exposure;; as well as examination of multiple COVID-19 outcome indicators spanning infection risk, symptomatology, recovery metrics, and mortality that provided a panoramic perspective on nutrition’s potential multi-faceted influences throughout the clinical spectrum. Main limitations include the preponderance of observational evidence inherently restricting causal inferences [44], the reliance of all studies on self-reported dietary assessment tools susceptible to recall biases and measurement errors [45]. Some studies had modest sample sizes, potentially compromising statistical power and generalizability. A notable geographical imbalance exists in the current research, with a lack of studies from Africa and the Caribbean. This gap limits our understanding of diet-COVID-19 relationships across diverse global contexts and may stem from disparities in research funding or infrastructure. Prospectively designed longitudinal analyses robustly controlling for key confounders are urgently needed to improve the evidence base and inform targeted dietary interventions.

## Conclusion

This scoping review synthesized evidence that high-quality, anti-inflammatory dietary patterns like the Mediterranean and DASH diets could mitigate COVID-19 severity and adverse outcomes, likely through modulating immunity and inflammation. However, a key gap is the reliance on observational studies, precluding causal inferences. Future research should prioritize prospective studies and randomized trials evaluating multi-faceted COVID-19 outcomes, mechanistic explorations of how dietary components modulate immune pathways, and potential synergies between optimal nutrition and other lifestyle factors. Integrating nutrition into pandemic preparedness strategies holds promise for safeguarding population resilience against future viral outbreaks.

## Data Availability

No datasets were generated or analysed during the current study.
